# Whipple's Pancreatoduodenectomy in the Background of Chronic Liver Disease (CLD): An Institutional Experience

**DOI:** 10.1155/2021/4848380

**Published:** 2021-12-02

**Authors:** Samrat Ray, Suvendu S. Jena, Amitabh Yadav, Sri Aurobindo Prasad Das, Naimish N. Mehta, Samiran Nundy

**Affiliations:** Sir Ganga Ram Hospital, New Delhi, India

## Abstract

**Introduction:**

Whipple's pancreatoduodenectomy (PD) is a formidable operation, associated with a high risk of morbidity and mortality. In the setting of an underlying chronic liver disease, the incidence of complications and mortality increases manifold. *Patients and Outcomes*. Of the 112 Whipple's PD performed between 2018 to 2020 at a high-volume HPB and liver transplant centre, 4 patients underwent the surgery in the background of an underlying chronic liver disease (CLD). All except one were performed in Child's A cirrhotics. There was a single 30-day mortality in this series of 4 patients that occurred in the background of Child's B cirrhosis. On follow-up at 1 year, there was one more mortality in the series, owing to liver decompensation following chemotherapy.

**Conclusion:**

Judicious preoperative selection criteria, adequate preoperative nutritional and physiological optimisation, and prudent weighing of risk vs. benefit of undergoing Whipple's PD in periampullary malignancies in the setting of CLD are the major determinants of the surgical outcome.

## 1. Introduction

Chronic liver disease (CLD) has traditionally been regarded as a contraindication to most major gastrointestinal (GI) and hepatopancreaticobiliary (HPB) procedures because there is, in some reports, a mortality of as high as 35% in these patients. Even in patients with normal liver function, pancreatoduodenectomy (PD) which is the treatment of choice for resectable periampullary malignancies has a morbidity of over 40% and a mortality of nearly 5–8%, in highly specialised tertiary-care centres [1, 2]. Apart from the intraoperative difficulties encountered during the surgery such as bleeding from dilated venous collaterals resulting from portal hypertension and coagulopathy from liver dysfunction, there may be difficult planes of dissection due to previous surgery and anaesthetic challenges. PD is also associated with a high risk of postoperative morbidity in the form of anastomotic leaks and hepatic decompensation [2].

Between January 2018 to December 2020 at a high-volume tertiary-care GI, HPB, and liver transplant centre in New Delhi, India, we operated on 4 patients with periampullary malignancies who also had CLD and retrospectively attempted to elucidate the surgical management of these patients who also had chronic liver disease and reviewed the published literature in an attempt to provide some guidelines for an experienced HPB surgeon.

## 2. Patients and Outcomes

Out of a total of 112 Whipple's pancreatoduodenectomy performed at our centre during these three years, there were 82 (73%) males and 40 females (38%) who had a mean age of 57 years (range 31–69 years). Of them, 3 patients had chronic liver disease (CLD) at the time of presentation and 1 was found to have features of CLD intraoperatively. Overall, 6 (5.3%) patients died 5 with normal livers and one case who had Child's B CLD. Their average length of postoperative hospital stay was 12 days (8–24).

The aetiology of CLD was Non-Alcoholic Steatohepatitis (NASH) related in all but one (Case 3). The worst MELD score noted in the series was 19 (Case 4). The preoperative fitness was assessed using the ECOG performance score/ASA scoring by the anaesthesiologist as well. Whipple's PD was performed in all for malignancies (adenocarcinoma) diagnosed preoperatively by EUS-guided biopsy. Three had some other comorbidities such as diabetes mellitus. Preoperative stenting was carried out in Case 2 in view of his bilirubin levels (>15 mg/dl) and in the other patient (Case 3) due to poor nutrition with decompensated CLD. None of the patients received any form of neoadjuvant chemo- or radiotherapy (Table 1).

Table 2 summarises the trend of laboratory variables in the postoperative period in comparison to the preoperative (baseline) value. Case 3 exhibited features of pancytopenia (related to CLD with hypersplenism) in the preoperative period, which was optimised to an adequate level before surgery. However, there was persistent decline in the haemoglobin and platelet counts noted in the postoperative period (surrogate markers of CLD decompensation and sepsis). The serum albumin levels also showed a similar trend in this patient, with persistent decline after POD 5, despite intravenous support.

**Figure 1:**
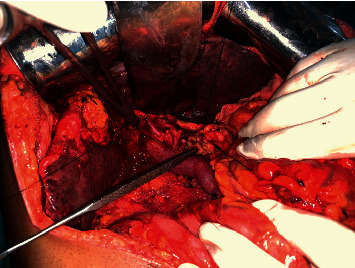
Intraoperative image showing grossly steatotic appearance of the liver with the transected pancreas (probe into the pancreatic duct) and hemostatic forceps probed into the common hepatic duct.

The operative duration ranged between 370 mins (Case 4) to 480 mins (Case 1) (Table 3). The four cases were performed by different senior surgeons of the same unit, following a uniform protocol of pancreatoduodenectomy. The intraoperative blood loss was aimed to be kept low to minimise the chances of decompensation and ranged from 700 ml to 900 ml. A single-loop reconstruction of the pancreatic-jejunal anastomosis with the duct to mucosa technique was performed in all but one (Case 4), where the isolated loop (Machado's) technique was used. Pancreatic dissection was deemed challenging in patients with liver disease owing to the presence of peripancreatic collaterals in 2 of the 4 cases (Case 1 and 3). There was no significant vascular event noted in any of the 4 cases. The liver was cirrhotic in two patients (Cases 1 and 3) and steatotic in the other two (Cases 2 and 4) (Figure 1). There was moderate ascites present in Case 3.

Table 4 summarises the postoperative outcome in the 4 patients. All required blood product transfusion in the postoperative period, the maximum (7 units packed cells with 8 units fresh frozen plasma) being required in Case 3. Local complications in the form of a clinically significant pancreatic leak were noted in 2 of the 4 cases (Cases 2 and 3), one of whom (Case 2) required placement of a percutaneous drain under USG guidance. One patient (Case 1) developed delayed gastric emptying on POD 5, with no evidence of any leak or collection (as documented by a CT of the abdomen on POD 5), which was managed with prokinetic drugs (metoclopramide and erythromycin started on POD 5). Case 3 developed severe ascites with features of hepatic encephalopathy (elevated ammonia levels) from POD 6, along with clinically a major pancreatic leak, eventually culminating into severe sepsis, disseminated intravascular coagulopathy (DIC), and multiorgan failure and died on POD 9. The other 3 patients were discharged after a postoperative stay of 10 days. Of the 3 patients, 1 (Case 4) was readmitted after 3 months of undergoing surgery with features of encephalopathy and decompensation related to CLD (probably secondary to two cycles of gemcitabine-based chemotherapy) and eventually died in the same admission after a hospital stay of 45 days. Case 1 and 2 have been followed up till 18 months after surgery and have shown no signs of recurrence or metastasis.

## 3. Discussion

The presence of underlying CLD in a patient with pancreatic carcinoma poses a formidable challenge to the surgeon planning a pancreatoduodenectomy (PD). The biggest dilemma is the “toss-up” situation between considering the risks of major surgery in a patient with CLD (documented mortality >30%) vs. the mortality associated with the primary malignancy and its effects on his or her quality of life. There have been very few studies in the literature assessing the impact of cirrhosis on the outcome of PD [3, 4]. One of the earliest reports came from a study by Artinyan et al. in 2012 who concluded that cirrhosis increased the perioperative morbidity and mortality in patients undergoing major GI oncological procedures [5]. The major challenges faced in patients with cirrhosis range in the intraoperative period from an increased likelihood of blood loss (due to underlying coagulopathy or massive collateral bleeding due to underlying portal hypertension) to encephalopathy and other haemodynamic alterations due to the anaesthetic drugs. In the postoperative period, decompensation in the form of intractable ascites, gastrointestinal bleeding, an increased risk of anastomotic dehiscence (poor healing), and resultant local and systemic sepsis account for the major causes of morbidity and mortality. Warnick et al. compared the outcome of pancreatic resection in patients with underlying cirrhosis against a matched control set of patients and reported a significantly high incidence of complications (47% vs. 22%; *P*=0.035) and reoperations (34% vs. 12%; *P*=0.03) [6]. In another recent study by El Nakeeb et al. conducted in 67 (Child's A and early B) out of 442 patients undergoing PD, the intraoperative blood loss and transfusion requirements were found to be significantly higher in the cirrhotic group [7]. The authors also reported a higher incidence of pancreatic fistula, wound complications, and haemorrhage in the cirrhotic group. Their duration of postoperative stay was also longer in the cirrhotic. Sethi et al. performed a retrospective analysis of the outcomes in 4 patients with operable pancreatic tumours and well-compensated CLD (CTP 5-6) over a 6-year period at a high-volume centre [8]. They reported a favourable short-term outcome in all their patients, with no requirement of blood transfusion, minimal intraoperative blood losses, and no postoperative hepatic decompensation. One death was reported at 18 months due to disease recurrence. Similar results were reported by the French group on a large multicentre study in 2015 [9]. Another group from Spain compared the outcome of PD in cirrhotics (*n* = 15) vs. noncirrhotics (*n* = 30) and reported a postoperative morbidity of nearly 60% in the former and also a higher duration of postoperative stay (25 ± 19 days) [10]. Findings of the studies are summarised in Table 5. However, they reported no difference in the rate of complications between the cirrhotic and noncirrhotic groups, as well as no difference in the rate of haemorrhage and fistulae. In the present case series over a period of 3 years at a high-volume HPB and transplant centre, we also reported findings similar to the studies mentioned above in terms of complications. However, a notable difference was observed in the incidence of immediate postoperative and long-term complications in our series. Factors such as difference in ethnicity and nutritional status between the West and the East could be an attributable factor [11]. The high incidence of decompensation and pancreatic leaks could be due to the already diminished healing and nutritional capacity of the patients, compounded by chronic alcohol intake (Case 3). Measures such as preoperative TIPSS have been suggested by few in patients with ascites to have a favourable outcome [12]. The single long-term mortality seen in the present series could be attributed to the effect of chemotherapy on underlying cirrhosis (Case 4).

Based on the survey of the existing literature and the findings of the current study, the authors would propose the following recommendations for performing Whipple's PD in the setting of underlying CLD:

1. **Judicious patient selection**: Child's A or early Child's B; ECOG performance 0 or 1.
2. **Adequate preoperative optimisation**: ascites, cholangitis, and nutritional rehabilitation are addressed. The waiting period should be at least 2-3 weeks after biliary stenting (avoid pancreatitis and bleeding). Options of TIPSS are considered for refractory portal hypertension.
3. **Hepatology and liver transplant critical care back-up**: it is ideal to be performed in centres with a team experienced in managing postoperative care of liver transplant recipients.

## 4. Conclusions

PD is a feasible option in patients with periampullary malignancy with underlying CLD. However, meticulous case selection based on Child's status, nutritional parameters, and blood indices and surgical expertise of the centre (high-volume HPB and transplant) could be the way forward to deal with such complicated case scenarios. The risk of mortality due to CLD must be weighed against the poor QoL and complications and mortality secondary to periampullary malignancy, and the same be explained to the patient.

## Figures and Tables

**Table 1:** Demographic and preoperative variables.

Variables	Case 1	Case 2	Case 3	Case 4
Age (yrs)	62	69	31	66
Gender	M	M	M	F
Preop diagnosis	Adenocarcinoma	Adenocarcinoma	Adenocarcinoma	Adenocarcinoma
Location of lesion	Ampulla	Head of the pancreas	Distal CBD	Head of the pancreas
Comorbidity (others)	Diabetes mellitus	Diabetes mellitus	None	Diabetes mellitus, hypertension
CLD aetiology	NASH	NASH	Alcohol	NASH
Worst Child's status (CTP score)	A (6)	A (6)	B (9)	A (6)
MELD score (worst)	17	18	19	16
Performance status (ECOG)	1	1	1	1
Fibroscan (kPa)	38.7	41.2	45.7	20.7
UGI endoscopy	Early oesophageal varices	Early portal hypertensive gastropathy	Large oesophageal varices	No varices
Preop cholangitis	Absent	Absent	Absent	Absent
Preop stenting	Not performed	Performed	Performed	Not performed
Preop neoadjuvant therapy	None	None	None	None

**Table 2:** Laboratory variables.

Variables	Case 1	Case 2	Case 3	Case 4
Haemoglobin	gm/dl			
Preop	11.2	12.1	9.5 (supp)	8.7
POD 1	9.2	11.1	7.2	8.1
POD 5	10	10.8	6.4	9.7

Platelet count	^*∗*^10^6^/cumm			
Preop	1.26	1.57	0.8	3.28
POD 1	1.1	1.81	0.6	2.55
POD 5	1.2	2.2	0.4	1.45

Albumin	gm/dl			
Preop	3.2	3.5	2.5 (supp)	2.35
POD 1	2.8	3.1	1.6	2.2
POD 5	3.1	3.2	1.5	2.1

Bilirubin	mg/dl			
Preop	5.49	17.4	2.35	9.18
POD 1	5.5	16.2	2.2	7.86
POD 5	3.1	4.1	1.1	2.2

ALT	U/L			
Preop	56	316	55	67
POD 1	67	164	40	68
POD 5	65	91	189	51

INR	—			
Preop	1.5	1.09	2.1	1.6
POD 1	1.1	0.9	1.7	1.2
POD 5	0.9	0.9	2.2	1.1

**Table 3:** Intraoperative variables.

Variables	Case 1	Case 2	Case 3	Case 4
Pancreatic parenchyma	Soft	Firm	Soft	Firm
Peripancreatic collaterals	Present	Absent	Present	Absent
PD diameter (mm)	3	4	2	12
Total blood loss (ml)	900	700	700	750
Operative time (mins)	480	435	440	370
Pancreaticojejunal anastomosis	Single loop; duct to mucosa	Single loop; duct to mucosa	Single loop; duct to mucosa	Isolated loop; duct to mucosa
Liver morphology	Micronodular	Diffusely steatotic	Micronodular	Steatotic
Ascites	Mild	Absent	Moderate	Absent

**Table 4:** Postoperative outcome.

Variables	Case 1	Case 2	Case 3	Case 4
Postop ICU stay (days)	2	2	3	2
Postop blood transfusion (units)	2	1	8	2
Local complications	Delayed gastric emptying (POD 5)	PJ leak (clinically significant) POD 6	PJ leak (clinically significant) POD 5; Delayed extraluminal haemorrhage (POD 8)	None
Systemic complications	None	Chest infection	Hepatic encephalopathy; Ascites; Chest infection	None
Postop hospital stay (days)	9	10	10	9
Clavien–Dindo grading	II	IIIa	V	I
Biospy (tumor stage)	T2N0M0	T1N0M0	T3N2M0	T3N1M0
Margin status (R)	R0	R0	R0	R0
Mortality (30 days)	No	No	Yes (POD 9)	No
Postoperative adjuvant therapy	None	None	NA	Yes (gemcitabine-based chemotherapy)
Follow-up mortality	No	No	NA	Yes (3 months after operation due to CLD decompensation)

**Table 5:** Review of the literature: Whipple's PD in the setting of CLD (comparative studies).

Authors (country)	Year	Sample size	Morbidity	Mortality
Warnick et al. [6] (Germany; single centre)	2011	32 cirrhotics (2 Child's B); 32 noncirrhotics	47% vs. 22% (*P* < 0.001)	3% (Child's A) vs. 100% (Child's B)

El nakeeb et al. [8] (Egypt; single centre)	2013	67 cirrhotic (4 Child's B); 375 noncirrhotic	20% vs. 10% (*P* < 0.02)	9.5% (Child's A) vs. 50% (Child's B)

Regimbeau et al. [9] (France; multicentre)	2015	35 cirrhotics (11 Child's B)	79% vs. 43% (*P*=0.002); 100% for Child's B	4% (Child's A) vs. 66% (Child's B)

Busquets et al. [10] (Spain; single centre)	2016	15 cirrhotics (0 Child's B); 30 noncirrhotics	73% vs. 53% (*P*=0.51)	0%

## Data Availability

Data are available on request.
